# Variation of *Neisseria gonorrhoeae* Lipooligosaccharide Directs Dendritic Cell–Induced T Helper Responses

**DOI:** 10.1371/journal.ppat.1000625

**Published:** 2009-10-16

**Authors:** Sandra J. van Vliet, Liana Steeghs, Sven C. M. Bruijns, Medi M. Vaezirad, Christian Snijders Blok, Jésus A. Arenas Busto, Marcel Deken, Jos P. M. van Putten, Yvette van Kooyk

**Affiliations:** 1 Department of Molecular Cell Biology and Immunology, VU University Medical Center, Amsterdam, The Netherlands; 2 Department of Infectious Diseases and Immunology, Utrecht University, Utrecht, The Netherlands; Northwestern University Feinberg School of Medicine, United States of America

## Abstract

Gonorrhea is one of the most prevalent sexually transmitted diseases in the world. A naturally occurring variation of the terminal carbohydrates on the lipooligosaccharide (LOS) molecule correlates with altered disease states. Here, we investigated the interaction of different stable gonoccocal LOS phenotypes with human dendritic cells and demonstrate that each variant targets a different set of receptors on the dendritic cell, including the C-type lectins MGL and DC-SIGN. *Neisseria gonorrhoeae* LOS phenotype C constitutes the first bacterial ligand to be described for the human C-type lectin receptor MGL. Both MGL and DC-SIGN are locally expressed at the male and female genital area, the primary site of *N. gonorrhoeae* infection. We show that targeting of different C-type lectins with the *N. gonorrhoeae* LOS variants results in alterations in dendritic cell cytokine secretion profiles and the induction of distinct adaptive CD4^+^ T helper responses. Whereas *N. gonorrhoeae* variant A with a terminal *N*-acetylglucosamine on its LOS was recognized by DC-SIGN and induced significantly more IL-10 production, phenotype C, carrying a terminal *N*-acetylgalactosamine, primarily interacted with MGL and skewed immunity towards the T helper 2 lineage. Together, our results indicate that *N. gonorrhoeae* LOS variation allows for selective manipulation of dendritic cell function, thereby shifting subsequent immune responses in favor of bacterial survival.

## Author Summary

*Neisseria gonorrhoeae* is a species of Gram-negative bacteria responsible for the sexually transmitted disease gonorrhea. Although effective antibiotic treatments are available, little is known about the host immune response to this pathogen. Here, we analyzed three well-characterized gonococcal variants that only differ in the outer sugar present on the lipooligosaccharide of the bacteria. We found that human dendritic cells use different receptors, including the C-type lectins MGL and DC-SIGN, to detect the three *N. gonorrhoeae* phenotypes. Dendritic cells carrying the MGL and DC-SIGN receptor were present in the human genital tissues, the site of gonoccocal invasion. DC activation with the gonococcal variants resulted in different cytokine secretion profiles and alterations in the subsequent adaptive T cell response. In particular, LOS containing a terminal *N*-acetylgalactosamine induced more T helper 2-type responses, which are unfavorable for clearing the bacteria. Our data provide new insights into the pathogenesis of *N. gonorrhoeae* and suggest that variation of lipooligosaccharide glycosylation enables to bacteria to selectively subvert host immune defense mechanisms.

## Introduction

*Neisseria gonorrhoeae* (gonococci, GC) is the causative agent of gonorrhea, one of the main sexually transmitted diseases. Global incidence has been estimated at 62 million infected people annually [1]. Although gonorrhea can remain asymptomatic, complications of the disease include pelvic inflammatory disease with a subsequent risk of infertility, and invasive and potentially life-threatening disseminated gonoccocal infection. Furthermore, gonorrhea is believed to predispose individuals to HIV-1 and *Chlamydia trachomatis* infection [2],[3].

GC colonize and invade the genital mucosal sites. The interaction between GC and the epithelial layer has been well characterized and involves the interaction of several GC virulence factors, including pili, porin and Opa proteins, to their respective receptors. Whereas GC pili may adhere to CD46 [4], the PorB porin attaches to heat shock protein Gp96 and the scavenger receptor SREC [5]. The opacity (Opa) proteins are divided into two major classes which adhere to either syndecans or CD66 family members [6].

After traversion of the epithelial cell barrier GC enter the submucosa, where they first encounter cells of the immune system, such as dendritic cells (DCs). DCs are the most potent antigen presenting cells of the immune system, capable of linking innate and adaptive immune responses [7],[8]. Immature DCs reside in all peripheral tissues where they act as sentinels to screen their surroundings for incoming pathogens. Upon microbial encounter immature DCs undergo a transitional process termed maturation, which involves migration to draining lymph nodes, upregulation of costimulatory molecules and the secretion of pro-inflammatory cytokines. In the lymph node mature DCs activate naïve T cells, thereby inducing adaptive immunity. In contrast to GC-epithelial interaction, little is known about the recognition of GC by DCs.

To detect pathogens DCs are equipped with a vast array of pattern recognition receptors (PPRs), including the Toll-like receptors (TLRs) and the C-type lectin receptors (C-type lectins) [9],[10]. In contrast to lipopolysaccharides (LPS) from enteric bacteria, GC express lipooligosaccharide (LOS), lacking the O-antigen. LOS can act as a GC virulence factor through recognition of its lipid A part by TLR-4/MD2 complex. The carbohydrate moiety of GC LOS is subject to phase variation, creating a GC population with different terminal carbohydrate residues on their LOS [11],[12]. These carbohydrate moieties may serve as ligands for sugar-dependent receptors, such as the C-type lectin DC-SIGN. Increasing evidence indicates that TLR and C-type lectin signaling cooperate and influence each other, thereby ensuing and controlling immune responses [13],[14]. Thus, variation of the terminal sugar of GC LOS could have a major impact on how GC modulates DC function.

**Figure 1:**
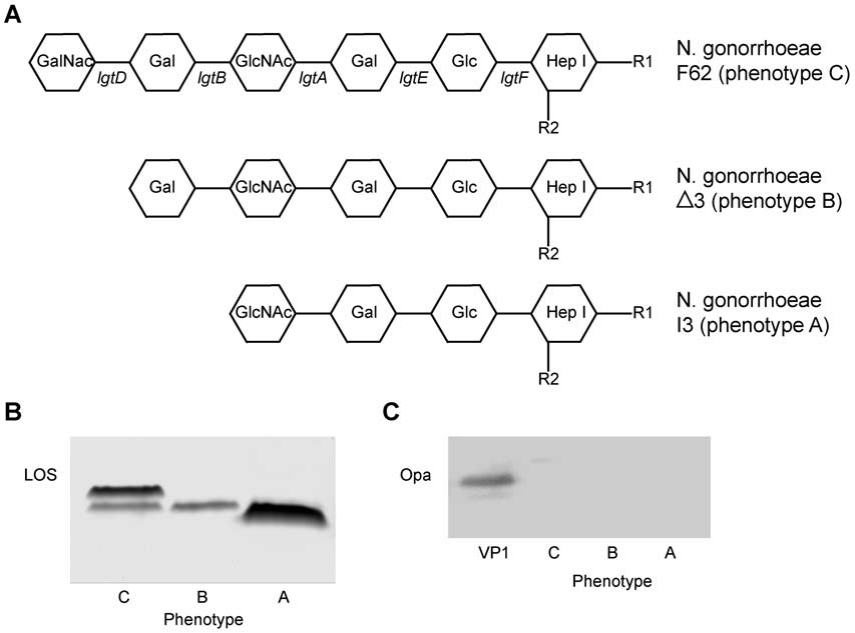
Characteristics of the *N. gonorrhoeae* F62 LOS phenotypes. (A) Schematic representation of the LOS strucuture of theisogenic LOS mutants of GC F62 (phenotype C) and its derivatives Δ3 (phenotype B) and I3 (phenotype A) that contain deletions within the glycosyltransferase genes *lgtD* and *lgtB* respectively. Figure adapted from [15]. (B) LOS silverstaining of tricine SDS-PAGE separated proteinase K digests of the different F62 phenotypes. (C) Western blot of whole cell lysates of the F62 variants incubated with the Opa-protein specific monoclonal antibody 4B12/CII. A lysate of the Opa-expressing strain *N. gonorrhoeae* strain VP1 served as a positive control.

We investigated three well-defined GC variants (Figure 1A), which only differ in their terminal LOS glycosylation [15], on DC recognition and their immunomodulatory properties. These variants are derived from GC strain F62 that contains LOS with mainly terminal *N*-acetylgalactosamine (GalNAc) residues. After inactivation of the glycosyltransferases genes *lgtD* and *lgtB* stable LOS variants were obtained that exhibit a terminal galactose and *N*-acetylglucosamine (GlcNAc), respectively. *In vivo* this variation occurs via slipped-stand mispairing of polynucleotide tracts within the *lgtD* gene, leading to exposure of a terminal GalNAc when the *lgtD* gene is functionally present and a terminal galactose residue when the gene is switched off [16]. The terminal galactose can serve as substrates for α2-3-sialylation rendering the bacteria resistant to complement killing [17],[18]. Thus, modification of the terminal galactose moiety appears to be of vital importance to the bacteria to subvert host defense systems.

Already in 1991 Schneider *et al.* demonstrated that when healthy volunteers were challenged with GC strain MS11 variant A (equivalent to the F62 variant B used in this study) the majority of the recovered variants expressed variant C (equivalent to F62 phenotype C) [19]. Their results indicate that GC LOS variants containing a terminal GalNAc residue have a selective advantage compared to variants ending in a terminal galactose. GC with phenotype C may have an enhanced ability to colonize or colonize epithelial cells, but may also posses immune evasive properties that explain the preferential outgrowth of this variant.

Here we demonstrate that the C-type lectin macrophage galactose-type lectin (MGL) is the DC-expressed receptor for *N. gonorrhoeae* strain F62 phenotype C and that recognition of the B variant is not C-type lectin-mediated. Furthermore, we confirm and extend on previous findings showing that the C-type lectin DC-SIGN interacts with the GC variant A [20],[21]. Although all GC variants induced equal DC maturation, phenotypic differences in the DC cytokines profiles and subsequent T cell polarization were observed based on the differential C-type lectin usage of the GC phase variants. Together these results provide a possible molecular mechanism contributing to the reported *in vivo* GC behavior [19] and indicate that variation of the GC LOS glycosylation interferes with the hosts ability to eradicate this pathogen.

## Results

### GC LOS glycosylation variants differentially interact with human DCs

To study the immunogenicity of GC that differ in their terminal LOS structure, we employed three well-characterized and stable non-piliated *N. gonorrhoeae* F62 variants [15]. The representative LOS glycan structures are depicted in Figure 1A. Variant B lacks the terminal GalNAc found in the F62 phenotype C and instead displays a terminal galactose. Variant A carries a terminal GlcNAc residue. Tricine SDS-PAGE confirmed the reported electrophoretic mobility of the LOS variants (Figure 1B). Phenotype C carries mainly LOS with a terminal GalNAc residue and a minor component with a terminal Gal residue [22],[23]. Western blotting of the variants showed equal Opa protein-negative phenotypes, excluding GC interactions with CD66 receptors (Figure 1C).

**Figure 2:**
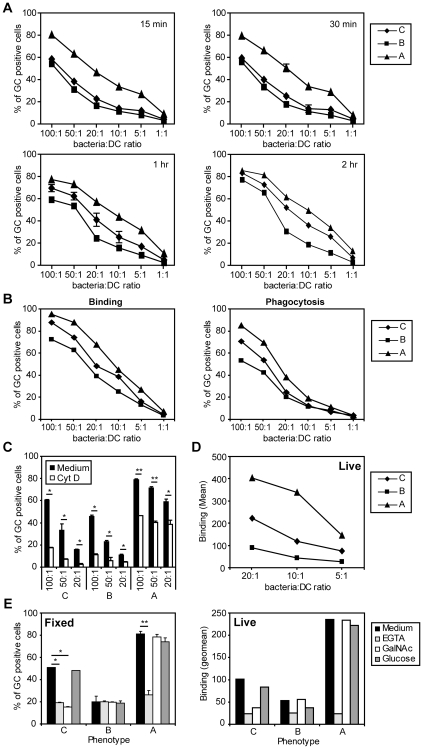
Differential binding of GC glycosylation variants to DCs. (A) Time course of dose dependent binding of FITC-labeled, fixed GC to immature DCs as analyzed by flow cytometry. GC were incubated for indicated time points at indicated bacteria∶DC ratios. Binding was significantly different for all phenotypes (P<0.05), except for the difference in binding of variants C and D after 15 minutes. (B) Binding and phagocytosis of FITC-labeled, fixed GC to immature DCs was measured by flow cytometry. GC were incubated for 1 hour at indicated bacteria∶DC ratios. Binding and phagocytosis was significantly different for all phenotypes (P<0.05). (C) Phagocytosis of fixed GC (1 hour at indicated bacteria∶DC ratios) can be blocked by the addition of the actin-polymerization inhibitor Cytochalasin D (10 µM). Depicted are the average+SD of triplicates. Cyt D, cytochalasin D (D) Dose dependent binding of FITC-labeled, live GC to immature DCs as analyzed by flow cytometry. GC were incubated for 1.5 hours at indicated bacteria∶DC ratios. (E) Binding of both fixed (left) and live (right) GC phenotypes A and C (1.5 hours at a bacteria∶DC ratio of 20∶1 for fixed and 10∶1 for live GC) to DCs is blocked by 10 mM EGTA. Binding of GC variant C but not A to DCs is inhibited by 100 mM of GalNAc. Depicted are the average+SD of three independent donors for the fixed GC and the average of two independent donors for the live GC. Significant differences: * P<0.05, ** P<0.01, *** P<0.001.

Bacteria were FITC-labeled to assess their interaction with human immature DC (Figure S1). Incubation of immature DC with the GC variants with the different LOS phenotypes at different bacteria∶ DC ratios yielded high level binding for variant A, intermediate binding levels for phenotype C and the lowest binding for the B variant (Figure 2A). These differences in interaction were mainly visible at prolonged incubation times of 1 and 2 hours (Figure 2A). Bacterial phagocytosis was analyzed by flow cytometry in the presence of trypan blue. Trypan blue quenches all extracellular fluorescence, allowing for the measurement of intracellular bacteria. All variants were phagocytosed at similar kinetics as observed in the binding experiments (Figure 2B and two additional donors in Figure S2), indicating that phagocytosis rate is largely determined by the total number of bacteria bound to the DC. In the presence of cytochalasin D (Cyt D), a known inhibitor of actin polymerization, phagocytosis of fixed GC was significantly blocked after 1 hour incubation, proving that the DCs actually internalized the GC (Figure 2C). Similar results were obtained after 2 hours of phagocytosis (data not shown). Our results with fixed GC were confirmed using live GC phenotypes, which were also bound by immature DC in a dose-dependent manner (Figure 2D). Similar to fixed GC, live GC of phenotype A bound at higher levels than variant C. Live GC of variant B showed the weakest binding to immature DCs.

To investigate the nature of the DC receptors involved, binding experiments were performed in the presence of the Ca^2+^-chelator EGTA, a well-known blocker of C-type lectin function. Binding of fixed GC phenotypes A and C was largely Ca^2+^-dependent and thus likely mediated by C-type lectins (Figure 2E, left panel). The residual binding, equivalent to variant B binding levels, could not be blocked by EGTA and may be conferred by other non-C-type lectin receptors. The addition of free GalNAc, but not control glucose monosaccharides reduced *N. gonorhoeae* phenotype C binding to variant B levels, indicating that a GalNAc-specific C-type lectin is responsible for binding of variant C (Figure 2E, left panel). Similar results were obtained for live FITC-labeled GC of phenotype C (Figure 2E, right panel).

### The C-type lectin MGL is a receptor for *Neisseria gonorrhoeae* phenotype C

One possible candidate C-type lectin is the asialoglycoprotein receptor (ASGP-R) which has a known specificity for galactose/GalNAc and is present on urethral epithelial cells where it facilitates entry of GC that express the complementary glycan structures [24]. However, this C-type lectin is not expressed on DCs. DCs do express the GalNAc-specific C-type lectin MGL [25]. We hypothesized that this lectin might be the major receptor for GC phenotype C.

**Figure 3:**
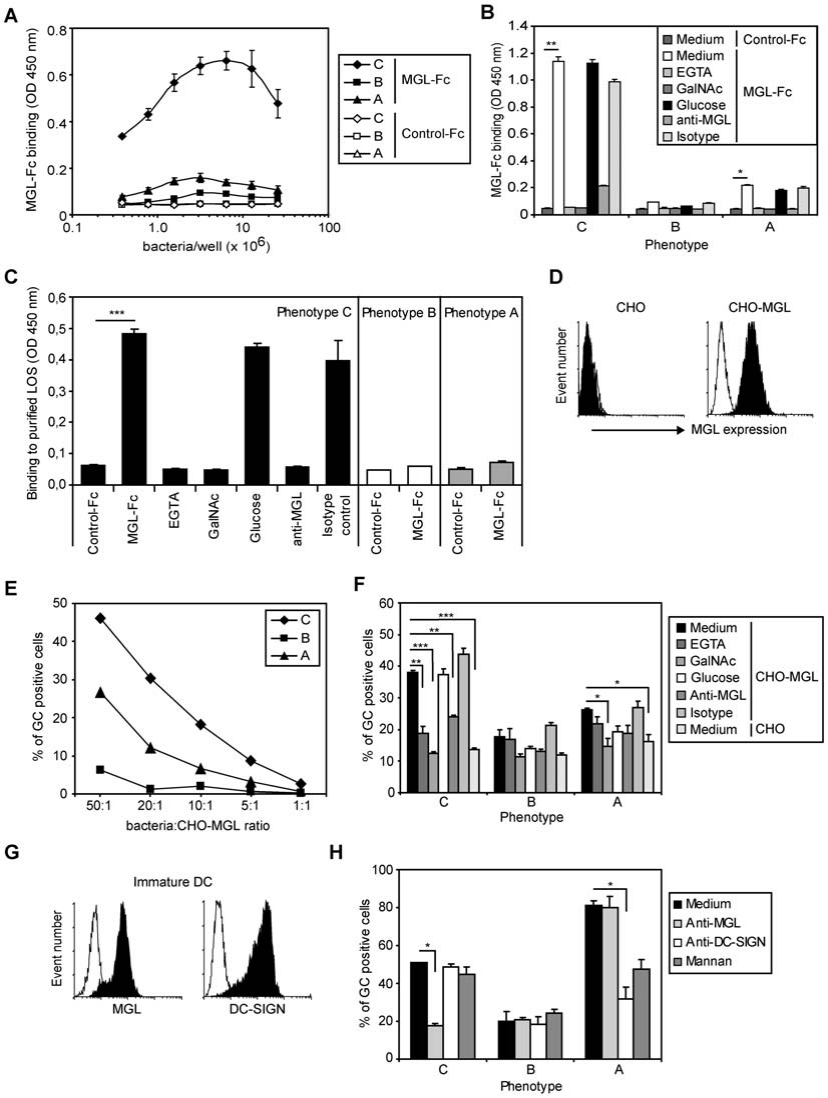
The C-type lectin MGL is a receptor for *N. gonorrhoeae* phenotype C. (A) Dose-dependent binding of MGL-Fc to coated GC. Depicted is the average±SD of triplicates. (B) MGL-Fc binding to GC phenotype C can be blocked by 100 mM of EGTA, 20 µg/ml of anti-MGL antibodies and the addition of free GalNAc monosaccharides (100 mM). GC were coated at 2.10^6^ bacteria/well and MGL-Fc binding was determined by an ELISA-based assay. Depicted is the average±SD of triplicates. (C) MGL-Fc binding to purified LOS from phenotype C can be blocked by 100 mM of EGTA, 20 µg/ml of anti-MGL antibodies and the addition of free GalNAc monosaccharides (100 mM). GC LOS was coated at a concentration of 10 µg/ml and MGL-Fc binding was determined by an ELISA-based assay. Depicted is the average±SD of triplicates. (D) Expression of MGL on parental and MGL-transfected CHO cells. Open histograms represent the isotype control and filled histograms represent the MGL staining. (E) CHO-MGL transfectants were incubated with FITC-labeled GC at indicated bacteria to CHO-MGL ratios for 30 minutes at 37°C and analyzed by flow cytometry. (F) Binding of FITC-labeled GC to CHO and CHO-MGL cells (30 minutes at a bacteria∶CHO-MGL ratio of 20∶1) was determined by flow cytometry in the presence or absence of EGTA, anti-MGL antibodies or free GalNAc monosaccharides. Depicted is the average±SD of triplicates. (G) Expression of MGL and DC-SIGN on immature monocyte-derived DCs. Open histograms represent the isotype control and filled histograms represent the MGL and DC-SIGN staining. (H) Binding of FITC-labeled GC phenotype C (1.5 hours at a bacteria∶DC ratio of 20∶1) to DCs is blocked by an anti-MGL antibody. Binding of GC variant A to DCs is blocked by an anti-DC-SIGN antibody (AZN-D1) and by the DC-SIGN specific ligand mannan (25 µg/ml). Depicted is the average±SD of triplicates. All results are representative of three independent experiments. Significant differences: * P<0.05, ** P<0.01, *** P<0.001.

A fusion protein of the extracellular domains of MGL coupled to the human IgG1 Fc tail strongly recognized GC variant C in a dose-dependent manner in an ELISA-based assay (Figure 3A). MGL-Fc also directly bound to purified LOS from variant C (Figure 3C). In contrast, GC and LOS from variants A and B only bound at background levels. MGL-Fc binding to GC phenotype C or purified LOS derived from this variant was blocked by the addition of the Ca^2+^-chelator EGTA, free GalNAc monosaccharides and anti-MGL antibodies, but not by glucose or isotype control antibodies, confirming the specificity of this interaction (Figure 3B and 3C).

Next, we investigated the recognition of GC phenotype C by cells that express the MGL protein, such as CHO-MGL transfectants and human monocyte-derived DCs (Figures 3D and 3G). CHO-MGL cells bound GC variant C with high affinity in a dose-dependent manner, while variant A adhered at low level and variant B was not recognized (Figure 3E). The interaction of MGL with GC phenotype C was specific, as shown by the inhibitory action of EGTA, free GalNAc monosaccharides and anti-MGL antibodies (Figure 3F). Immature DCs expressed both the C-type lectins MGL and DC-SIGN (Figure 3G). Binding of GC variant C to DCs was blocked to variant B levels using specific anti-MGL antibodies, whereas binding of phenotype A was inhibited by pretreating the DCs with anti-DC-SIGN antibodies or the DC-SIGN ligand mannan (Figure 3H and [20],[21]). Although we observed some residual binding of CHO-MGL to variant A (Figure 3F), in DCs binding of this phenotype was largely DC-SIGN-mediated. The residual binding is probably independent of the LOS carbohydrate variation as all variants bind equally well to DC after inhibiting C-type lectin function. Thus, immature DCs interact with GC variants C and A mainly through the lectins MGL and DC-SIGN, respectively.

### MGL and DC-SIGN expressing cells are present in male and female genital tissues

**Figure 4:**
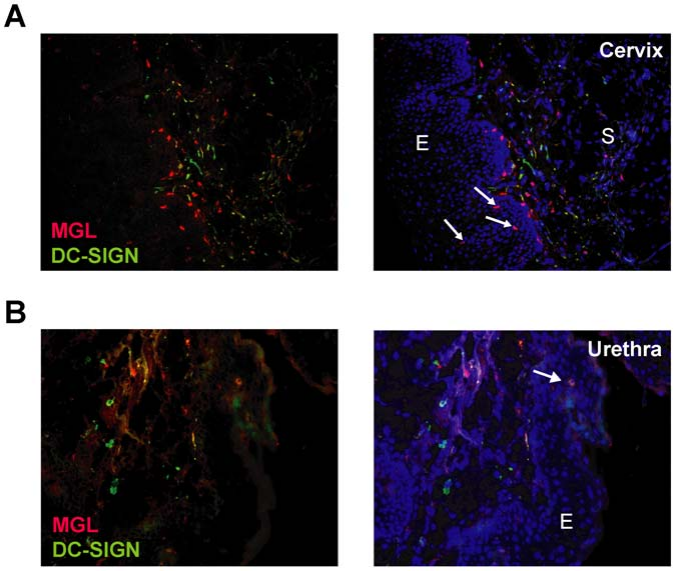
MGL and DC-SIGN are present at the site of GC entry. Expression of MGL (red) and DC-SIGN (green) in cryosections of human ectocervix (A, left) and penile urethral tissue (B, left). Nuclei are stained with Hoechst (blue, right pictures). MGL positive cells in the epithelial layer are indicated by the white arrows. Original magnification 200×. E, epithelium; S, sup-epithelial tissue.

If the C-type lectins MGL and DC-SIGN are physiological important receptors that play an active role in the pathogenesis of GC, these proteins should be expressed at the site of GC entry, the female cervix and the male penile urethral tissue. DC-SIGN^pos^ cells have been shown to be present in cervix [26], however it is currently unclear whether MGL^pos^ cells are located there as well. Therefore, we stained healthy human urethra and cervix with antibodies to DC-SIGN and MGL. MGL^pos^ cells were clearly visible just beneath and in the epithelial layers of both tissues (Figure 4). These results are in contrast to human skin, where MGL^pos^ cells are exclusively localized in the dermis [27]. A subset of the MGL^pos^ cells in the cervix co-expressed the marker CD1a (data not shown), confirming this subset to be DCs. DC-SIGN expression was confined to the sub-epithelium on a subset of cells that did not co-express the MGL molecule. Thus, both MGL and DC-SIGN were expressed at the site of GC entry and could thus participate in the recognition of this pathogen by local antigen presenting cells.

### *Neisseria gonorrhoeae* modulates DC cytokine profiles

**Figure 5:**
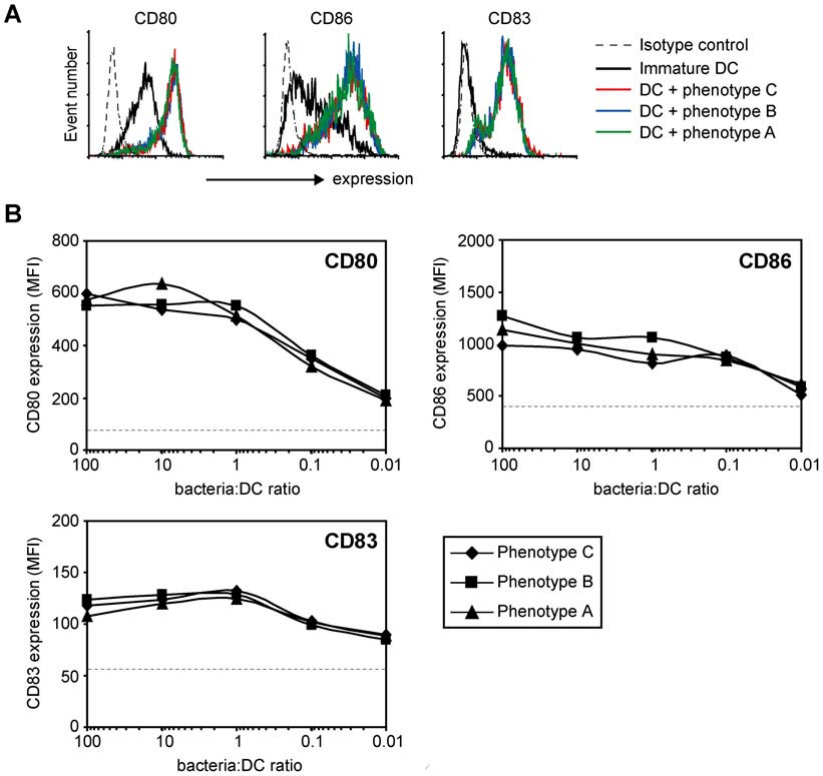
GC LOS variation does not influence expression of DC maturation markers. Immature DC were incubated with unlabeled fixed GC at a bacteria∶DC ratio of 100∶1 (A) or in a dose-dependent range (B). After 16 hours of stimulation the expression of DC maturation markers CD80, CD83 and CD86 was measured by flow cytometry. The dashed line indicates the expression level in immature DCs. One out of four independent donors is shown.

Having established that both MGL and DC-SIGN are present at the site of infection, we investigated the functional consequences of the *N. gonorrhoeae*-DC interaction. Immature DCs were incubated with the different unlabeled fixed (to prevent bacterial overgrowth) GC phenotypes and analyzed for expression of maturation markers and secretion of pro- and anti-inflammatory cytokines. All GC strains were potent inducers of DC maturation as visualized by the increased expression levels of CD80, CD83 and CD86 even at very low bacteria to DC ratios (Figure 5), suggesting that LOS variation does not affect the expression of DC maturation markers.

**Figure 6:**
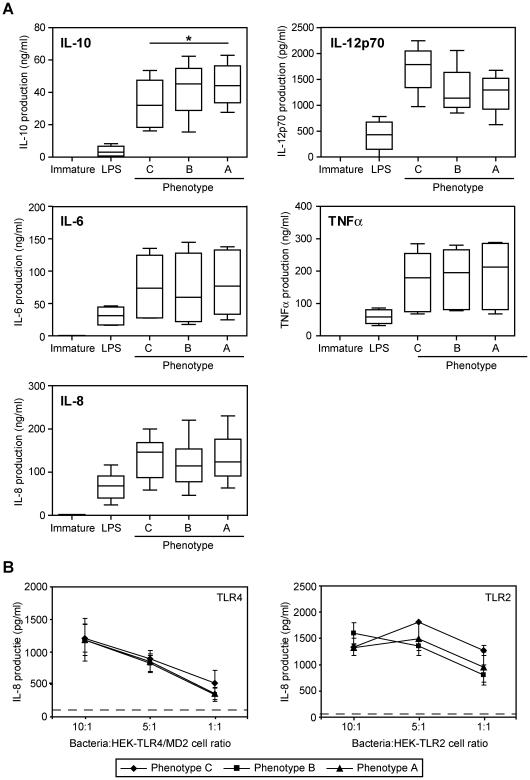
DCs incubated with GC variant C secrete significantly less IL-10 compared to DCs stimulated with variant A. (A) Immature DCs were incubated with unlabeled fixed GC at a bacteria∶DC ratio of 100∶1 for 16 hours. Supernatants were harvested and cytokine production was measured by ELISA. *E. coli* LPS (10 ng/ml) was included as a positive control. The box plots represent the combined data of six independent donors. * indicates a significant difference in IL-10 production by DCs stimulated with GC phenotype C compared to DCs stimulated with GC phenotype A (p<0.05). (B) All phenotypes activate TLR2 and TLR4 equally as measured by IL-8 release by HEK-TLR2 and HEK-TLR4/MD2 reporter cells. Depicted is the average±SD of triplicates. Dashed line indicates the basal IL-8 levels of unstimulated cells.

Strikingly, cytokine secretion was influenced by variation of LOS. Immature DCs pulsed overnight with GC at a bacteria∶DC ratio of 100∶1 produced extremely high levels of IL-10, IL-12p70, IL-6, IL-8 and TNFα (Figure 6A). IL-10 production was only significantly decreased in DCs incubated with GC phenotype C compared to DCs incubated with GC variant A (Figure 6A). The slight increase in IL-12p70 secretion by DCs pulsed GC phenotype C was not statistically significant. IL-6, IL-8 and TNFα levels remained unchanged (Figure 6A). We could not detect any IL-17 production by GC-stimulated DCs, eliminating the possibility that the GC aspecifically bind the antibodies used in this assay (data not shown). The observed effects were not due to differences in TLR2 or TLR4 triggering, as all strains induced equal TLR activation in HEK-TLR2 or HEK-TLR4 reporter cells (Figure 6B). Thus, variation of terminal glycans on GC LOS directly affects cytokine secretion by DCs, potentially modifying the induction of adaptive immune responses.

### Induction of T helper responses by GC-matured DC

**Figure 7:**
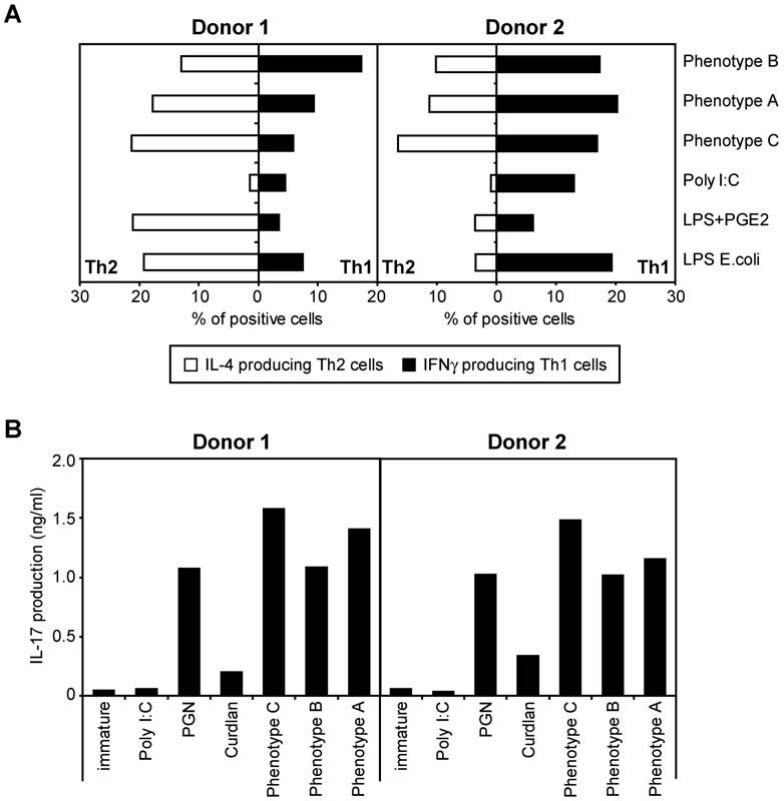
GC are potent inducers of human CD4^+^ T helper responses. (A) T helper responses induced by GC phenotype C-stimulated DCs are skewed towards Th2. Naïve CD4^+^ T cells were cocultured with DCs stimulated with unlabeled fixed GC at a bacteria∶DC ratio of 100∶1. After 14 days intracellular IL-4 and IFNγ production by the T cells was analyzed on a single cell basis by flow cytometry. *E. coli* LPS (100 ng/ml), LPS+PGE_2_ (100 ng/ml and 10 µg/ml respectively) and Poly I∶C (20 µg/ml) were included as positive controls of mixed, Th2 and Th1 skewing, respectively. Results of two representative donors out of five are shown. (B) DCs stimulated with GC phenotype C induce slightly stronger Th17 responses. CD4^+^ T cells were cocultured with DCs stimulated with unlabeled fixed GC at a bacteria∶DC ratio of 100∶1. After 4 days IL-17 production was measured in the supernatant by ELISA. Poly I∶C was used as a negative control, peptidoglycan (PGN, 10 µg/ml) and the β-glucan curdlan (10 µg/ml) were included as positive controls. Results of two independent donors are shown.

DCs play a key role in shaping adaptive immunity. Upon pathogen encounter DC undergo a phenotypic transformation termed maturation, followed by migration to the lymph node, where the mature DCs prime naïve T cells to become effector T cells. The multitude of innate signals received from the pathogen largely determines the nature of the adaptive T cell response. To mimic this, we primed DCs with the different fixed GC variants and subsequently co-cultured these DCs with naive T cells to analyze T helper (Th) cell differentiation. Th1 and Th2 polarization was defined by the relative shifts in IL-4 (for Th2) and IFNγ (for Th1)-producing T cells. In this assay, relative to the LPS-matured DC, Poly I∶C and *E. coli* LPS supplemented with PGE_2_ served as controls for Th1 and Th2 skewing, respectively. Interestingly, DCs pulsed with GC phenotype C had an increased ability to functionally polarize Th2 cells (Figure 7A), irrespective whether the donors were more prone to induce Th1 (donor 2) or Th2-type responses (donor 1). Even though the number of Th1 cells remained unchanged, in donor 2 an increase in Th2 cells was observed using DCs stimulated with variant C. DCs incubated with GC phenotype B predominantly generated Th1 T cells, whereas T cells activated by DCs co-cultured with GC variant A displayed an intermediate phenotype (Figure 7A).

Human DCs readily promote the development of Th17 cells from memory T cells [28]. Th17 responses are important for clearance of bacterial infections and are initiated after activation of the intracellular pattern recognition receptors NOD2 or dectin-1 [29],[30]. Natural ligands for NOD2 and dectin-1 comprise peptidoglycan (PGN) and curdlan, respectively. PGN fragments are also released during GC growth suggesting that *in vivo N. gonorrhoeae* might also induce Th17-type immunity [31]. Compared to the positive controls *S. aureus* PGN and curdlan, all GC variants triggered robust Th17 differentiation in human T cells as measured by the specific IL-17 production in these cells. In both donors, the strongest Th17 polarization was observed with the DCs incubated with GC phenotype C (Figure 7B). Together, our data indicate that *N. gonorrhoeae* has the potential to actively modify its LOS to manipulate DC-induced adaptive immune responses.

## Discussion

The lack of a suitable non-primate animal model for *Neisseria gonorrhoeae* (gonococci, GC) has hampered our knowledge on the immunological events following infection with this obligate human pathogen. By relying on *in vitro* epithelial cell culture models the mechanisms of GC entry and invasion have been extensively studied (reviewed in [32]). Here, we provide more insight into how the human immune system and specifically dendritic cells deal with naturally occurring LOS variants of *N. gonorrhoeae*. DCs are in general the first cells of the immune system to encounter local pathogens and they are instrumental in the induction of subsequent innate and adaptive immune responses [7]. Our results indicate that GC can target different C-type lectin receptors on DCs by varying its LOS. We confirm previous results from Zhang *et al.* which demonstrate the interaction of DC-SIGN with *N. gonorrhoeae* variant A [20],[21]. C-type lectins and especially DC-SIGN have already been shown to interact with a vast array of pathogens including several bacterial species [33]–[36]. Known pathogens that engage MGL include the helminth parasite *Schistosoma mansoni* and several members of the *filoviridae* family [25],[37]. Yet, no bacterial ligands have been described so far for the human MGL receptor. C-type lectins constitute the main receptors for GC on DCs, as only some residual binding to DC was observed for GC phenotype B or the variants A and C after blocking MGL and DC-SIGN function respectively, to DCs. Other known receptors for GC surface factors such as pili and Opa proteins on DCs include members of the CD66 family, CD46 and CR3 [4], [38]–[40]. Targeting of these receptors may have additional immunomodulatory effects. We used non-piliated GC that lack detectable Opa protein expression to specifically dissect the DC-LOS interaction. Opa-deficient GC have previously been reported to still maximally adhere to human DCs [21]. We also considered siglec receptors that specifically recognize α2,3-sialylated LOS [41] as potential receptors. However, these receptors are mainly present on macrophages and mature DC and low to absent on immature DC [42],[43].

Our results suggest that based on the differential receptor engagement, GC may influence subsequent immune responses initiated by DCs through variation of their LOS, a feature never before reported for *N. gonorrhoeae*. C-type lectins are already implicated in homeostatic control by DC through the binding and capture of self-glycoproteins [44]. In the steady state, the antigen-handling capacity of C-type lectins on DCs can maintain peripheral tolerance through deletion of self-reactive T cells and the generation of regulatory T cells [45]. As several GC LOS glycan structures mimic human membrane glycosphingolipids, it is tempting to speculate that GC exploit the regulatory pathways induced by these C-type lectins in favor of their own survival. Protective immune responses to *N. gonorrhoeae* are generally T helper 1 (Th1)-type in nature, leading to the production of complement-fixing antibodies, as patients with defects in the complement pathways often suffer from recurrent and disseminated gonoccocal infections [46]. Here, we show that GC phenotype C induces phenotypic changes in DCs leading to alterations in the cytokines secreted by the matured DCs and more pronounced Th2 and Th17 responses. The combined expression of both GalNAc and galactose on the terminal end of LOS phenotype C (as shown in Figure 1C and [22],[23]), indicates that our results may even underestimate the effects a terminal GalNac residue could have on human DCs. To our knowledge induction of Th17 cells by *N. gonorrhoeae* has not previously been reported, but the related bacterium *N. meningitidis* also instructs the polarization of Th17-type T cells [28]. Th17-type immunity has been shown to facilitate the destruction and clearance of fungi and extracellular bacteria [47]. Skewing towards Th2 could result in a decreased production of complement-fixing antibodies, which may delay bacterial eradication by the host defense. GC have developed a variety of mechanisms to avoid complement-mediated killing, the so-called serum-resistance. Stable serum resistance can be mediated through sialylation of the LOS oligosaccharide and through binding of the porin protein to the complement regulatory protein factor H [48]. We suggest that deviation towards unfavourable T helper responses may be another, more indirect gonococcal immune evasion strategy.

We observed significantly increased IL-10 production by DCs stimulated with GC phenotype A. Several bacterial species, including *Mycobacterium tuberculosis* and *Helicobacter pylori*, target the C-type lectin DC-SIGN to selectively induce IL-10 secretion [33],[36]. Strikingly, these pathogens all inflict chronic infections, suggesting that the IL-10 may be important in this process, by creating an environment that supports pathogen survival while limiting tissue pathology. Therefore, LOS structures containing the terminal GlcNAc of the variant A glycan could aid the bacterium to establish latent infection. It has been suggested that only variation in lipid A moieties can influence proinflammatory cytokine production in human cell lines and primary monocytes [49],[50]. Yet, our results indicate that also the carbohydrate component of LOS can directly modulate DC responses, as the TLR2 and TLR4 activating potential was equal for all GC variants. This apparent discrepancy could be explained by C-type lectin expression levels, which are high on DCs and low to absent in human monocytes and epithelial or monocytic cell lines. Hence, C-type lectin-mediated signals would only be able to affect cytokine secretion in human DCs.

The high rate of re-infection suggests that *N. gonorrhoeae* possesses several strategies to actively suppress host adaptive immune reactions; however these mechanisms are only beginning to be unraveled. GC expressed Opa proteins can directly suppress T cell receptor signaling through engagement of coinhibitory CD66a/CEACAM1 receptor, thereby raising the threshold for CD4^+^ T cell activation and proliferation to activating stimuli such as IL-2 or CD3 ligation [51],[52]. The CD66a/CEACAM1-Opa interaction also impedes antibody production by promoting cell death in human B cells [53]. Overall, humoral immune responses to *N. gonorrhoeae* are relatively modest and our results suggest that in addition to killing B cells, GC may also be capable of influencing T helper responses to alter the isotype of the elicited antibodies. Recently, an influx of regulatory T cells was observed in a newly refined model of *N. gonorrhoeae* infection in 17β-estradiol treated mice [54],[55]. However, regulatory T cells were only detected at relatively late time points, suggesting that these suppressive T cells were newly induced or expanded shortly after inoculation of the bacteria. Next to immunity, DCs have also been shown to directly contribute to the expansion and differentiation of regulatory T cells through the expression of inhibitory molecules belonging to the B7 or ILT family or through the secretion of suppressive cytokines such as IL-10 [56]. These so-called tolerogenic IL-10DCs can generate antigen-specific regulatory T cells [57]. Strikingly, DCs stimulated with GC and especially GC phenotype A, secrete extremely high levels of IL-10. We speculate that the high IL-10 production might facilitate the formation of regulatory T cells, which could, during ongoing infection, limit pathogen-specific immune responses and thus contribute to successful colonization of the bacteria.

Based on our results, it can be argued that variation of the GC LOS glycan structures could have a functional relevance for bacterial survival within an infected individual. Different terminal glycans can target different C-type lectin receptors on human DCs thereby modifying DC function and possibly also host immune responses. LOS of variant A induces high IL-10 secretion by the DC, which might aid the outgrowth of regulatory T cells. Our data indicates that the terminal GalNAc residue, as found on LOS of GC phenotype C, skews T helper differentiation towards unfavorable Th2-type responses. This scenario of events collaborates the early findings of Schneider *et al.* that showed that active disease is associated with expression of longer LOS variants containing terminal GalNAc residues [19]. Modulation of the terminal galactose might therefore be important for bacterial pathogenesis in the host. Indeed, *in vivo* the terminal galactose is usually capped by either a GalNAc, which, based on our studies, might modulate DC function, or a sialic acid moiety, which confers serum-resistance.

In conclusion, our results extend the plethora of immune evasion mechanisms that have evolved within the *N. gonorrhoeae* species. The strategic bacterial intervention operating at multiple levels of the human immune system, including DCs, likely explains the maintained persistence of this bacterium in its obligatory human host.

## Materials and Methods

### Bacterial strains

*Neisseria gonorrhoeae* strain F62 and its derivatives Δ3 and I3 (structures shown in Figure 1) were generously provided by E.C. Gotschlich (Rockefeller University, New York, USA) [15]. Non-piliated strains were grown on GC agar base (Difco, Becton Diskinson France) supplemented with 1% Vitox (Oxoid, Basingstoke, UK) at 37°C in an atmosphere of 5% CO_2_ at 37°C. When appropriate, bacteria were inactivated in 0.5% paraformaldehyde in phosphate buffered saline (PBS) for 15 minutes followed by thorough washing in RPMI 1640 medium without phenol red (Invitrogen, Carlsbad, CA, USA). FITC-labeled bacteria were prepared by incubation of 2.10^9^ bacteria with 0.5 mg/ml of FITC (Sigma-Aldrich, St. Louis, MO, USA) for 20 minutes at 37°C followed by extensive washing. Bacterial suspensions with an optical density at 540 nm of 1, corresponding to 10^9^ bacteria/ml, were prepared in RPMI 1640 medium without phenol red. All bacteria were equally FITC-labeled as determined by FACS (Figure S1).

### Cells

Human immature monocyte-derived DCs were cultured for 4–7 days in RPMI 1640 medium (Invitrogen) containing 10% Fetal Calf Serum from monocytes obtained from buffy coats of healthy donors (Sanquin, Amsterdam, the Netherlands) in the presence of IL-4 and GM-CSF (500 U/ml and 800 U/ml respectively, Biosource, Camarillo, CA, USA). CHO cells were maintained in RPMI 1640 medium containing 10% Fetal Calf Serum. Stable CHO-MGL and CHO-DC-SIGN transfectants were regularly selected using 1 mg/ml Geneticin (Invitrogen). To check for C-type lectin expression, cells were incubated with primary antibody (AZN-D1 (DC-SIGN [58]), 18E4 (MGL [27]), 5 µg/ml), followed by staining with a secondary FITC-labeled anti-mouse antibody (Zymed, San Francisco, CA) and analyzed on a FACScan (BD Biosciences, San Diego, CA). For analysis of DC maturation, DCs were incubated for 30 minutes at 4°C with phycoerythrin-conjugated antibodies to CD83 (Beckman Coulter, Fullerton, CA), CD80 and CD86 (both from BD Biosciences) and analyzed on a FACScan.

### LOS isolation, electrophoresis, and Western blotting

The LOS phenotype of the GC was confirmed by tricine SDS-PAGE of proteinase K-treated bacteria and silverstaining as described [18]. Purified LOS was prepared as described [59]. Expression of Opa proteins was determined by SDS-PAGE and Western blotting using the Opa protein-specific monoclonal antibody 4B12.CII as previously described [60]. The gonococcal strain VP1 expressing Opa protein served as a positive control.

### Cellular binding and internalization assays

Binding experiments were conducted by incubating immature DCs, CHO parental or CHO transfectants with FITC-labeled GC at indicated bacteria∶DC ratios for indicated time points at 37°C in RPMI/0.5% BSA. Binding was analyzed by flow cytometry and is represented as the percentage of cells that have bound the fluorescent bacteria. Phagocytosis was determined in the presence of a 1∶10 dilution of Trypan Blue (0.4%) to quench the fluorescence of the extracellular bacteria [21]. Specificity of binding was assessed in the presence or absence of EGTA (10 mM), blocking antibodies (20 µg/ml), mannan (25 µg/ml, Sigma-Aldrich) or free GalNAc monosaccharides (100 mM). When appropriate, DCs were pretreated with 10 µM Cytochalasin D (to block actin polymerization, Sigma-Aldrich) for 30 minutes at 37°C. Cytochalasin D was present during the entire assay.

### MGL-Fc binding assays

GC or purified LOS were coated in PBS at indicated concentrations on NUNC maxisorb plates (Roskilde, Denmark) overnight at room temperature. Plates were blocked with 1% BSA and MGL-Fc was added (0.5 µg/ml) for 2 hours at room temperature in the presence or absence of 10 mM EGTA, 100 mM free GalNAc monosaccharides or 20 µg/ml mAbs. Binding was detected using a peroxidase-labeled anti-human IgG-Fc antibody (Jackson, West grove, PA). MGL-Fc and DCIR-Fc control, comprising of the extracellular domains of MGL and DCIR fused to the human IgG1 Fc tail, were generated as previously described [25],[34]. Fc-proteins were purified from the supernatant of CHO-transfectants using protA columns.

### Immunohistochemistry

Cryosections of healthy human tissues (7 µ) were fixed with 100% aceton and stained with primary antibodies to DC-SIGN and MGL (10 µg/ml) for 1 hour at 37°C. Sections were counterstained with isotype-specific Alexa488- or Alexa594-labeled anti-mouse antibodies (Molecular probes, Carlsbad, CA). Nuclei were visualized using Hoechst.

### Cytokine measurements

DC supernatants were harvested 24 hours after DC activation and frozen at −80°C until analysis. Cytokines were measured by ELISA with CytoSets™ ELISA kits (Biosource), according to the manufacturer's protocol. Human IL-12p70 detection was determined as previously described [61].

### TLR2 and 4 reporter assay

HEK293 cells stably expressing TLR2 or TLR4 and MD2 [62], a gift from D. Golenbock (University of Massachusetts Medical School, Worcester, USA), were stimulated with the GC at indicated bacteria∶cell ratios. After 24 hours, supernatants were analyzed for IL-8 production by ELISA according to the manufacturer's guidelines (Biosource/Invitrogen).

### DC driven polarization of T helper responses

The skewing of T helper 1 (Th1) and Th2 responses was determined as previously described [33]. Briefly, immature monocyte-derived DCs were stimulated with unlabeled formaldehyde fixed GC at a bacteria∶DC ratio of 100∶1. *E. coli* LPS (100 ng/ml, Sigma-Aldrich), LPS+PGE_2_ (100 ng/ml and 10 µg/ml respectively, Sigma-Aldrich) and Poly I∶C (20 µg/ml, Invivogen, San Diego, CA) were included as positive controls of mixed, Th2 and Th1 skewing, respectively. After 2 days, DCs were washed and incubated with allogeneic naïve CD4^+^ T cells (ratio 1∶10). In parallel, DCs were analyzed for maturation markers by flow cytometry. Untouched naïve CD4^+^ T cells were isolated using MACS isolation from PBMC by depleting all non-CD4^+^ and memory T cells (Miltenyi Biotec, Bergisch Gladbach, Germany). At day 5, IL-2 (10 U/ml) was added. At day 12 or 13, quiescent T cells were re-stimulated with 30 ng/ml PMA and 1 µg/ml ionomycin (both Sigma-Aldrich) for 6 h. After 1 hour 10 µg/ml Brefeldin A (Sigma-Aldrich) was added to the T cells. Single cell production of IL-4 and IFNγ was determined by intracellular flow cytometric analysis. Cells were fixed in 2% PFA, permeabilized with 0.5% saponin (Sigma-Aldrich) and stained with anti-human IFNγ-FITC and anti-human IL-4-PE (BD Biosciences).

T helper 17 (Th17) responses were determined as previously described [28]. Briefly, immature monocyte-derived DCs were stimulated with unlabeled formaldehyde fixed GC at a bacteria∶DC ratio of 100∶1. Poly I∶C (20 µg/ml) was used as a negative control, *Staphylococcus aureus* peptidoglycan (PGN, 10 µg/ml, Sigma-Aldrich) and the β-glucan curdlan (*Alcaligenes faecalis*, 10 µg/ml, Sigma-Aldrich) were included as positive controls for Th17 skewing. After 24 hours DCs were washed and incubated with allogeneic memory CD4^+^ T cells (ratio 1∶10). Untouched memory CD4^+^ T cells were isolated using MACS isolation from PBMC by depleting all non-CD4^+^ and naive T cells (Miltenyi Biotec, Bergisch Gladbach, Germany). At day 5, IL-17 production by the T cells was measured by ELISA according to the manufacturer's protocol (eBioscience, San Diego, CA).

### Statistical analysis

Data were analyzed for statistical significance using a two-tailed paired Student *t*-test. Cytokine levels were compared using ANOVA followed by Bonferroni's Multiple Comparison Test. All p-values<0.05 were considered significant.

### Accession numbers

MGL: CD301/*CLEC10A*, GenBank/EMBL NM_006344. DC-SIGN: CD209, GenBank/EMBL AF290886.

## Supporting Information

Figure S1 Representative FITC-labeling of GC. Bacteria were labeled with 0.5 mg/ml of FITC for 20 minutes at 37°C followed by extensive washing and analysis by flow cytometry. Open histograms represent unlabeled bacteria and filled histograms represent FITC-labeled baceria.
(0.10 MB TIF)

Figure S2 Differential binding of GC glycosylation variants to DCs. Binding and phagocytosis of FITC-labeled, fixed GC to immature DCs of donor B (A) and donor C (B) was measured by flow cytometry. GC were incubated for 1 hour at indicated bacteria∶DC ratios. Phenotype A was significantly different from variants B and C in both binding (P<0.01) and phagocytosis (P<0.05). Phenotype B was significantly different from phenotype C in binding (P<0.05) but not phagocytosis.
(0.30 MB TIF)
