# Supplementary figures and images for: Variation of Neisseria gonorrhoeae Lipooligosaccharide Directs Dendritic Cell–Induced T Helper Responses
Source: PLoS Pathog. 2009 Oct 16;5(10):e1000625. doi: 10.1371/journal.ppat.1000625 (PMC2757725; doi:10.1371/journal.ppat.1000625)

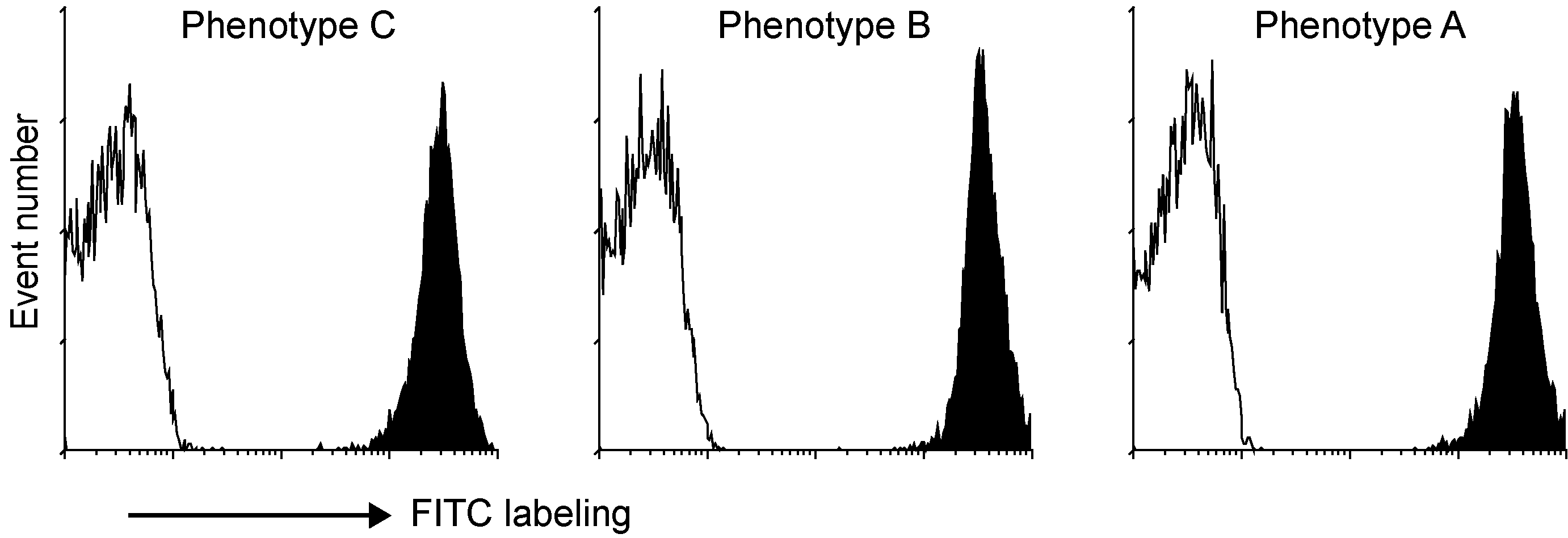

Supplement: Figure S1 — Representative FITC-labeling of GC. Bacteria were labeled with 0.5 mg/ml of FITC for 20 minutes at 37°C followed by extensive washing and analysis by flow cytometry. Open histograms represent unlabeled bacteria and filled histograms represent FITC-labeled baceria. (0.10 MB TIF) [file ppat.1000625.s001.tif]

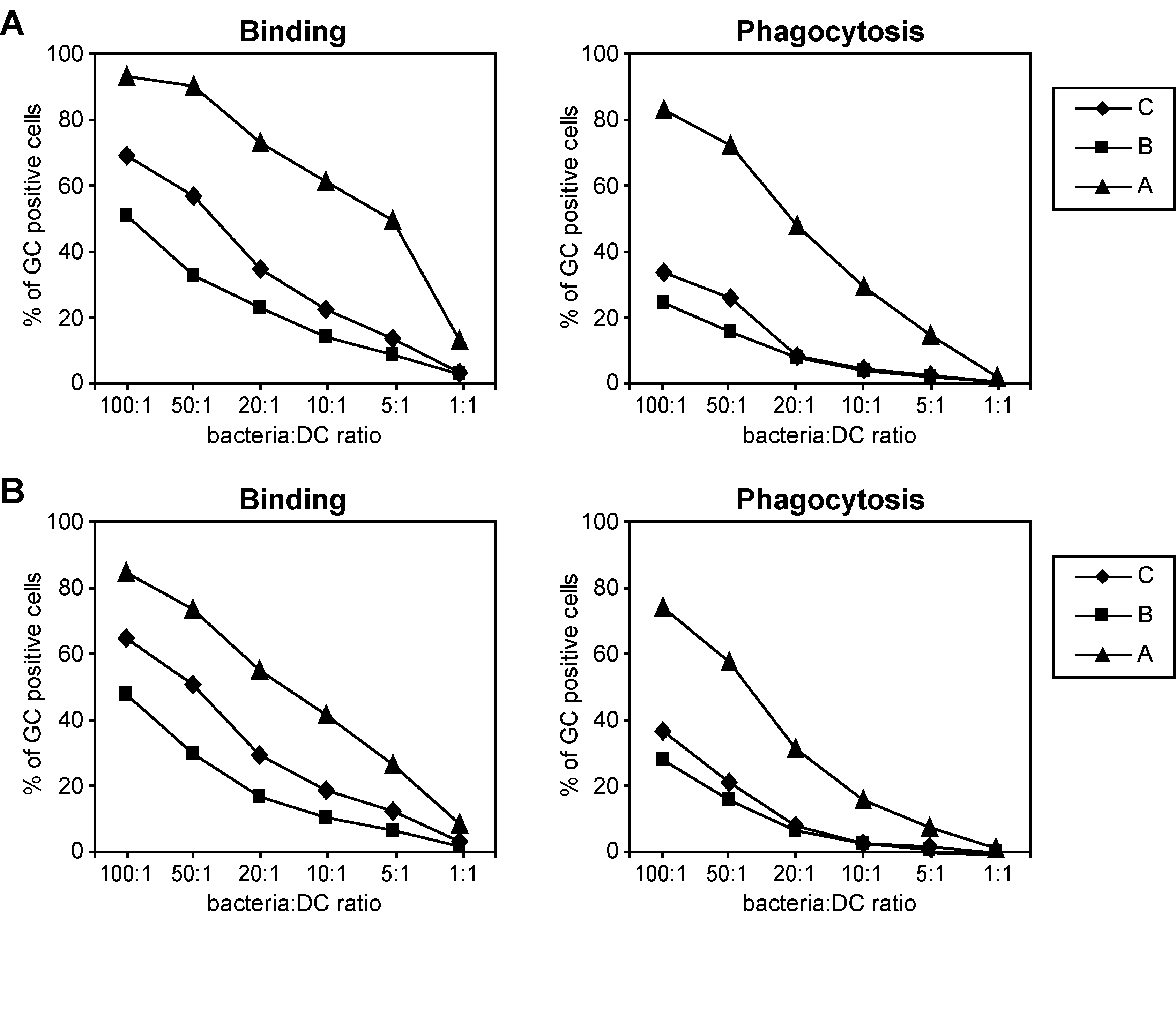

Supplement: Figure S2 — Differential binding of GC glycosylation variants to DCs. Binding and phagocytosis of FITC-labeled, fixed GC to immature DCs of donor B (A) and donor C (B) was measured by flow cytometry. GC were incubated for 1 hour at indicated bacteria∶DC ratios. Phenotype A was significantly different from variants B and C in both binding (P<0.01) and phagocytosis (P<0.05). Phenotype B was significantly different from phenotype C in binding (P<0.05) but not phagocytosis. (0.30 MB TIF) [file ppat.1000625.s002.tif]
